# Effect the Sintering Temperature on the Microstructure and Flexural Strength of ZrO_2_ Ceramics Produced by NanoParticle Jetting

**DOI:** 10.3390/ma18112605

**Published:** 2025-06-03

**Authors:** Youji Huang, Xiaorong Li, Hongyu Chen, Kun Ren, Huijun Guo, Huan Qi

**Affiliations:** 1 College of Mechanical Engineering, Zhejiang University of Technology, Hangzhou 310023, China; 211122020057@zjut.edu.cn; 2 Key Laboratory of Special Purpose Equipment and Advanced Processing Technology, Ministry of Education and Zhejiang Province, Zhejiang University of Technology, Hangzhou 310023, China; 3 Sinopec Ningbo Engineering Company Limited, Ningbo 315103, China; lixiaorong.snec@sinopec.com; 4 School of Mechanical Engineering, Suzhou University of Science and Technology, Suzhou 215009, China; renkun@usts.edu.cn; 5 Advanced Materials Additive Manufacturing Innovation Research Centre, Hangzhou City University, Hangzhou 310015, China

**Keywords:** zirconia ceramic, nanoparticle jetting, sintering temperature, flexural strength

## Abstract

Zirconia ceramics (ZrO_2_) have received significant attention due to their excellent mechanical properties and broad application prospects. Additive manufacturing, especially nanoparticle jetting (NPJ), offers a new approach for fabricating zirconia ceramics with complex geometries. However, the sintering process plays a crucial role in determining the final properties of these ceramics, and the effect of sintering temperature on NPJ printed zirconia ceramics remains to be fully understood. This study investigates the impact of sintering temperature on the properties of zirconia ceramics fabricated via NPJ. NPJ-printed ZrO_2_ green bodies were sintered at varying temperatures, and their phase composition, microstructure, and flexural strength were analyzed. Results show that as the sintering temperature rises from 800 °C to 1450 °C, the relative density of ZrO_2_ increases from 55.0% to 98.3%, and the flexural strength rises from 9.3 MPa to 356.1 MPa. The green body consists of monoclinic (m-ZrO_2_) and tetragonal (t-ZrO_2_) phases, with m-ZrO_2_ completely transforming into t-ZrO_2_ at 1000 °C. Grain size also increases with temperature. The improvement in zirconia’s flexural strength is primarily attributed to a combination of grain size and porosity. This research provides guidance for optimizing the sintering process of NPJ-printed ZrO_2_ ceramics.

## 1. Introduction

Zirconia ceramics, a typical oxide ceramic material, exhibits three crystal structures: monoclinic, tetragonal, and cubic, with densities of 5.65 g/cm^3^, 6.10 g/cm^3^, and 6.27 g/cm^3^, respectively [1]. These crystalline forms undergo phase transformations under specific temperature conditions. At room temperature, zirconia generally exists in the monoclinic phase, which transitions to the tetragonal phase at 1170 °C. Upon further heating to 2370 °C, the tetragonal phase transforms into the cubic phase [2]. Zirconia ceramics are characterized by their excellent mechanical properties (e.g., compressive strength of approximately 2000 MPa and fracture toughness of 5–10 MPa·m^1/2^ [3,4]). In addition, they exhibit high wear and corrosion resistance. Due to these properties, zirconia ceramics are widely used in aerospace, automotive manufacturing, biomedical, and other fields [5,6,7,8]. Meanwhile, as a biologically inert material, zirconia ceramic is non-toxic and exhibits excellent biocompatibility [9]. Its color and luster closely resemble those of natural teeth, thereby ensuring optimal natural aesthetics. Consequently, it has been extensively utilized as a restorative material within the oral cavity [10]. Among these, 3 mol% yttria-stabilized tetragonal polycrystalline zirconia ceramics (3Y-TZP), one of the most widely used ceramic materials in biomedicine, is extensively applied in the field for manufacturing dental restorations such as full crowns and implants [11,12]. In summary, zirconia, as an advanced ceramic material, offers a broad spectrum of applications due to its exceptional comprehensive properties.

Additive manufacturing (AM), also known as 3D printing, emerged in the late 1980s [13]. This technology builds parts through layer-by-layer material deposition. AM excels in fabricating components with highly complex geometries [14]. According to the international standard ISO/ASTM 52900:2021 [15], AM technologies are classified into seven categories: vat photopolymerization (VPP), material extrusion (ME), material jetting (MJ), binder jetting (BJ), sheet lamination (SL), powder bed fusion (PBF), and directed energy deposition (DED). Common AM techniques for zirconia ceramics include MJ [16,17,18], VPP [19,20], PBF [21,22,23], and FDM [24]. VPP employs UV light to selectively cure layers of photosensitive resin, constructing parts layer by layer. After curing each layer, the platform moves by a predefined thickness, repeating this process until the part is complete. The two main VPP techniques, stereolithography (SLA) and digital light processing (DLP) [25], differ primarily in their light sources and curing mechanisms. SLA uses an ultraviolet laser guided by a dynamic galvanometer mirror to cure the resin in a point-to-line-to-surface sequence based on the pre-designed cross-sectional geometry [26]. In contrast, DLP projects a full-layer cross-sectional image of UV light onto the resin surface using a digital micromirror device, curing the entire layer simultaneously [27]. VPP offers high printing accuracy and excellent surface quality, achieving a resolution exceeding 1 μm [28] and enabling the fabrication of intricate ceramic structures [29]. These advantages have established VPP as a widely employed technique for producing zirconia ceramic components [30]. Despite its benefits, challenges remain, including mismatches in photorefractive indices between ceramic and resin, and light scattering due to ceramic absorption. These issues can reduce printing precision and affect final product performance [7,31]. Additionally, high-temperature AM techniques such as PBF and FDM often lead to defects such as low molded density, surface roughness, and cracks. These challenges stem from zirconia’s intrinsic properties, including a high melting point, limited ductility, and poor thermal shock resistance [32,33,34]. In contrast, material jetting does not require the high temperatures involved in PBF and FDM processes. It combines thin printing layer thickness with high resolution, enabling high-precision printing [35,36,37,38]. This provides a new method for the additive manufacturing of zirconia ceramics. Nanoparticle Jetting (NPJ), as a prominent material jetting technology, demonstrates exceptional printing capabilities with achievable layer thicknesses of 5–10 µm and surface roughness values as low as Ra 3 µm. The process facilitates high-precision fabrication of complex architectures while simultaneously achieving a high green density and near-full sintered density [39]. Moreover, unlike the PBF process, the operator does not need to handle powder or resin during the NPJ printing process, reducing the risk of injury [40].

The sintering process involves complex microstructural and property evolution of materials, which is a key factor in enhancing the properties of ceramics [41]. The sintering parameters, including the sintering temperature, holding time, and heating rate, exert varying degrees of influence on the sintering properties of ceramics [42,43]. Currently, extensive research has examined microstructural evolution and mechanical property changes in zirconia ceramics during sintering, including densification behavior, shrinkage [44,45,46], sintering temperature effects on grain size [47,48,49] and mechanical properties [50,51,52]. For instance, Mazaheri et al. [46] investigated the effects of two different sintering methods on the grain size of zirconia ceramics. The results demonstrated that two-step sintering significantly reduces grain size compared to single-step sintering. Hu et al. [50] reported that increasing sintering temperature led to gradual decreases in porosity and average pore size, resulting in corresponding compressive strength enhancement. Ji et al. [51] studied sintering temperature effects on grain growth, phase composition and mechanical properties. Based on grain growth characteristics, the sintering process can be divided into three stages: early sintering, mid-sintering, and late sintering, and it was pointed out that most of the densification was completed in the mid-sintering stage.

However, studies on the sintering behavior of NPJ-printed zirconia ceramics remain limited. Yongduk Oh initially investigated the dielectric properties of NPJ-printed zirconia ceramics but did not address mechanical properties [53]. Later, Willems et al. [39] and Zhong et al. [40] examined the microstructure and mechanical properties of zirconia ceramics produced via NPJ. However, both studies employed a fixed sintering temperature of 1450 °C, leaving the effect of sintering temperature change on mechanical properties unexplored.

Therefore, this study investigates NPJ-printed zirconia ceramics sintered at different temperatures, discussing their phase composition, microstructure, and flexural strength. The aim is to explore the influence of sintering temperature on the flexural strength of these ceramics.

## 2. Materials and Methods

### 2.1. Green Body Printing and Sintering Process

Zirconia rectangular green bodies were prepared using an XJET Carmel 1400 3D printer (XJET, Rehovot, Israel). The composition of the zirconia ceramic slurry and support material is shown in Table 1, and the type of zirconia contained was 3 mol% yttria-stabilized zirconia. The incorporation of yttria elevates the free energy of the monoclinic phase while simultaneously reducing that of both tetragonal and cubic phases. This energetic modification reduces the phase transition temperature. This effect becomes more pronounced with increasing dopant concentration, ultimately stabilizing the tetragonal and even cubic phases at room temperature. As XJET’s state-of-the-art additive manufacturing system, the printer’s mask module is equipped with 24 print heads, each comprising 512 nozzles. During the printing process, the system’s 12,288 nozzles precisely deposit suspensions of model and support materials onto the build platform, ejecting up to 120 million droplets per second with high precision. This enables the efficient fabrication of products with highly complex structures. Among the 24 printheads, 12 are dedicated to depositing ZrO_2_ as the model material, while the other 12 are used for the support material. After deposition is complete, a heating system consisting of six halogen lamps and a heated tray generates temperatures ranging from 160 °C to 230 °C, evaporating the liquid components in the suspensions and leaving behind dense, ultrafine ceramic layers. A roller passes over the newly deposited layer for controlling the desired height. Subsequently, the build platform is lowered to initiate the subsequent deposition cycle.

**Figure 1:**
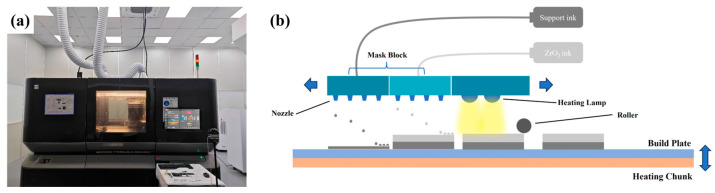
XJet Carmel 1400 3D printer: (**a**) photograph of the printer; (**b**) schematic diagram of the printing process.

The zirconia ceramic sintered bodies were designed with dimensions of 25 mm (X) × 5 mm (Y) × 2.2 mm (Z) and a print layer thickness of 10 μm. Following the completion of the print job, the removal of the support material was initiated. The green bodies were immersed in deionized water, resulting in the dissolution and subsequent removal of the support structure, which was made of soluble material. This process ultimately led to the detachment of the green bodies from the build platform. Figure 1 shows the physical drawing of the XJET Carmel 1400 and its schematic diagram of the printing process.

**Figure 2:**
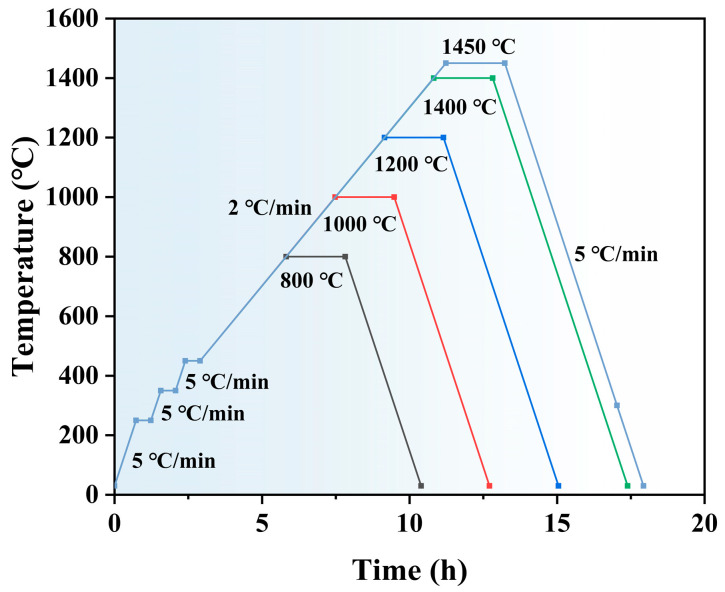
Debinding and sintering program for zirconia ceramics.

The sintering process was carried out using a 3D printed high-temperature sintering furnace (FMJ-42/17, Hefei Feishiruo Thermal Equipment Co., Ltd., Hefei, China) under atmospheric pressure. The specific sintering procedure was as follows: the temperature was increased from 30 °C to 250 °C at a heating rate of 5 °C/min and then held at 250 °C for 30 min for sufficient drying. Subsequently, the temperature was raised to 350 °C at the same rate for debinding, and the temperature was held for 30 min in order to facilitate a smooth transition in temperature. Then, the temperature was further increased to 450 °C at the same rate to ensure complete debinding. Following this, the temperature was increased to the final sintering temperature at a rate of 2 °C/min and maintained for 2 h. The results of previous experiments have indicated that the increase in relative density and flexural strength of NPJ-printed zirconia ceramics tends to slow down when the sintering temperature reaches 1400 °C. To further investigate the impact of elevated sintering temperatures on ceramic properties, the final sintering temperatures were set at 800 °C, 1000 °C, 1200 °C, 1400 °C, and 1450 °C, in accordance with references [37,38]. Subsequent to the sintering process, the sintered body was permitted to cool to ambient temperature with the furnace at a rate of 5 °C/min. This approach was adopted to avert the occurrence of cracking in the sintered body, which might be caused by an accelerated cooling rate. The sintering curve is shown in Figure 2.

### 2.2. Material Property Characterization

**Figure 3:**
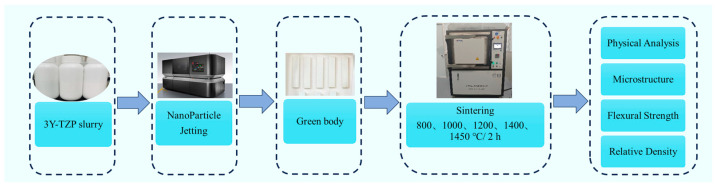
The experimental flowchart of this paper.

The phase composition analysis of the samples was conducted using an X-ray diffractometer (XRD, Bruker D8A A25 X, Billerica, MA, USA) with the following measurement parameters: Cu target, voltage of 40 kV, current of 40 mA, scanning range of 10° to 90°, step size of 0.02°, and scanning speed of 5.7°/min. The surface and cross-sectional microstructures of the samples were examined using a scanning electron microscope (SEM, Zeiss Gemini 360, Oberkochen, Germany). During SEM observation, the operating parameters were set as follows: an accelerating voltage of 10 kV, a beam current of 0.8 nA, a dwell time of 5 μs, and a magnification of 10,000×. Prior to surface observation, necessary pretreatment steps were performed. For samples sintered at 800 °C and 1000 °C, the surfaces were ground smooth and coated with a Pt conductive layer before observation. For samples sintered at 1200 °C, 1400 °C, and 1450 °C, the surfaces were polished to a mirror-like finish using diamond polishing paste, thermally etched at 100 °C below their sintering temperatures for 20 min and then coated with Pt for observation. For cross-sectional observation, the samples were directly coated with Pt and then examined. Sample mass was determined with an electronic balance (BSM-420.3, Shanghai Zhuojing Electronic Technology Co., Ltd., Shanghai, China). Statistical analysis of grain size and pore size was performed on surface SEM images using the ImageJ-win64 image analysis software version 2024. To ensure the reliability and representativeness of the statistical results, a minimum of 100 random measurements were taken, and the average of these measurements was calculated to determine the statistical outcomes of the feature sizes. The relative density of the samples was calculated based on their dimensions and mass, with the theoretical density considered to be 6.05 g/cm^3^ [39]. The flexural strength of the samples was determined through a three-point bending test, with a span length of 20 mm and a loading rate of 0.5 mm/min. In order to reduce the margin of error, five samples at each sintering temperature were selected for measurement. A schematic experimental flowchart for this paper is shown in Figure 3.

## 3. Results and Discussion

### 3.1. Density

**Figure 4:**
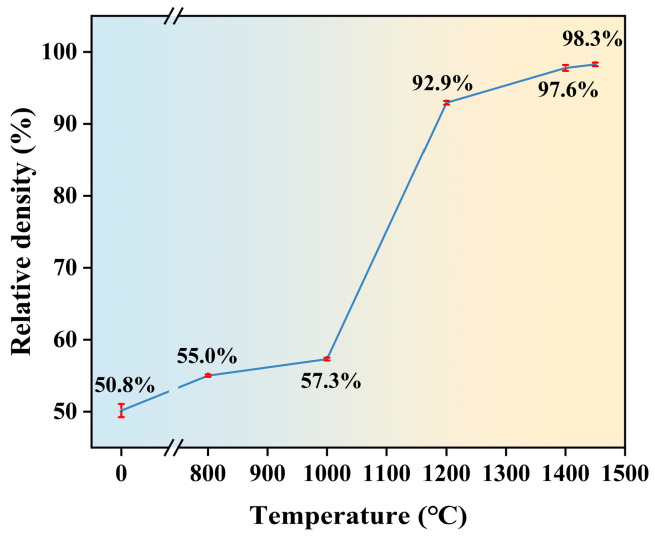
Relative density of zirconia ceramics at different sintering temperatures.

As demonstrated in Figure 4, the relative density of zirconia ceramics is shown to vary with sintering temperature. The figure illustrates that the relative density of the green body (i.e., the sintering temperature is 0 °C) is 50.8%. The relative density exhibits an S-shaped increasing trend as the sintering temperature is increased. Based on the rate of change in relative density, the sintering process can be divided into three stages. The first stage occurs at sintering temperatures from 800 °C to 1000 °C, where the relative density increases from 55.0% to 57.3%. Following this, the sintering temperature rises to the range of 1000 °C to 1200 °C, marking the second stage. During this phase, the relative density increases at an accelerated rate. By the end of the second stage, it reaches 92.9%, indicating that most densification occurs during this period. As the sintering progresses into the third stage, between 1200 °C and 1450 °C, the rate of change in relative density dramatically decreases. In particular, the relative density increased by a mere 0.7% within the temperature range of 1400 °C to 1450 °C, which indicates that the densification of zirconia ceramics is basically completed. According to the literature [54], Chen et al. prepared zirconia ceramics using digital light processing technology, and conducted sintering experiments in the temperature range of 1400–1520 °C. The results showed that the relative density of sintered zirconia ceramics could exceed 99% when the sintering temperature reached 1520 °C. The zirconia ceramics prepared by the nanoparticle jetting technique in this study achieved a relative density of 98.3% after sintering at 1450 °C. Although this value is slightly lower than the previous one, this difference is mainly due to the lower sintering temperature used in this study.

### 3.2. Phase Composition Analysis

**Figure 5:**
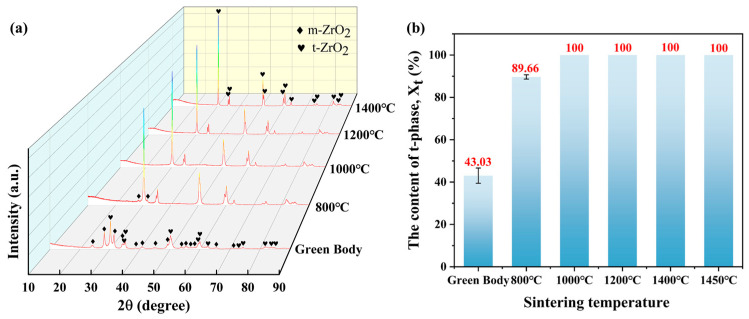
(**a**) XRD patterns and (**b**) phase composition proportions of ZrO_2_ ceramics in the green body and at different sintering temperatures.

To further determine the phase composition of the zirconia ceramics, the green body and zirconia ceramics, sintered at different temperatures, were subjected to X-ray diffraction (XRD) analysis, the results of which are presented in Figure 5a. As illustrated in the figure, the green body is composed of monoclinic (m-ZrO_2_) and tetragonal (t-ZrO_2_) phases. As the sintering temperature increased, the m-ZrO_2_ phase in the zirconia ceramics underwent a gradual transformation into the t-ZrO_2_ phase. The phase transition temperature of t-ZrO_2_ to c-ZrO_2_ in zirconia is approximately 2370 °C. In this study, the maximum sintering temperature was 1450 °C, and thus the generation of the c-ZrO_2_ phase was not detected in the samples prior to and following sintering. Additionally, to perform a quantitative analysis of the phase transformation during the sintering process, the method proposed in the reference [55] was used to determine the content of the two phases:

(1) $$X_{m}=\frac{I_{m\left(111\right)}+I_{m\left(\bar{1}11\right)}}{I_{m\left(111\right)}+I_{m\left(\bar{1}11\right)}+I_{t\left(101\right)}}$$

where $X_{m}$ is the content of m-ZrO_2_, $I_{m\left(111\right)}$ and $I_{m\left(\bar{1}11\right)}$ are the diffraction peak intensities of m-ZrO_2_ at the (111) and (−111) crystal planes, respectively, and $I_{t\left(101\right)}$ is the diffraction peak intensity of t-ZrO_2_ at the (101) crystal plane.

Since no c-ZrO_2_ was detected in any of the samples, the content of t-ZrO_2_ $X_{t}$ was calculated using the following formula:

(2) $$X_{t}=1-X_{m}$$

Figure 5b illustrates the content of t-ZrO_2_ in zirconia ceramics within the green body and at different sintering temperatures. The initial content of t-ZrO_2_ in the green body was determined to be 43.03%. With the gradual increase in sintering temperature, the content of t-ZrO_2_ in the sintered samples continued to rise steadily. At a sintering temperature of 800 °C, compared to the green body, the diffraction peak intensities of the (111) and (−111) crystal planes decreased significantly, while all diffraction peaks associated with m-ZrO_2_ disappeared. Simultaneously, the intensity of the (101) diffraction peak increased markedly, corresponding to a t-ZrO_2_ content of 89.66% at this stage. When the sintering temperature is further increased to 1000 °C, only t-ZrO_2_ diffraction peaks are observed in the XRD patterns, corresponding to a t-ZrO_2_ content of 100%. Further increasing the sintering temperature to 1450 °C results in the consistent observation of only t-ZrO_2_ diffraction peaks in the XRD patterns.

During the sintering process, the phase transition temperature of m-ZrO_2_ to t-ZrO_2_ in zirconia ceramics is about 1170 °C. However, in this paper, m-ZrO_2_ completely transformed into t-ZrO_2_ at 1000 °C. The reason for this phenomenon may be related to the Y_2_O_3_ doping in the raw materials.

The fundamental driving force for phase transitions in zirconia ceramics is the decrease in the free energy of the system. During the sintering process, the free energy of m-ZrO_2_, t-ZrO_2,_ and c-ZrO_2_ changes as the temperature increases. When the free energy of one phase is lower than that of the current phase, the system will spontaneously shift to a lower free energy phase to achieve thermodynamic stability. In this paper, zirconia powder stabilized with 3 mol% Y_2_O_3_ used as the raw material. Y_2_O_3_ is used as a dopant where the introduction of Y^3+^ is able to decrease the free energy of t-ZrO_2_ while increasing the free energy of m-ZrO_2_. This leads to a decrease in the free energy difference between the two phases and therefore a decrease in the driving force required for the phase transition during sintering, lowering the phase transition temperature from m-ZrO_2_ to t-ZrO_2_.

### 3.3. Microstructure and Flexure Strength

**Figure 6:**
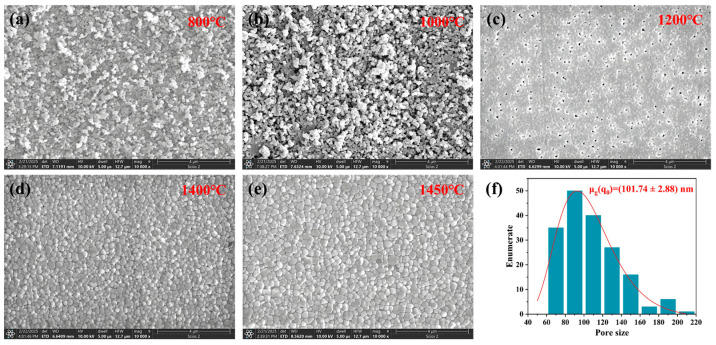
(**a**–**e**) SEM images of the surface of zirconia ceramics sintered at different temperatures; (**f**) the pore size distribution on the surface of zirconia ceramics sintered at 1200 °C.

The variation in the microstructure of the zirconia ceramic surface with temperature is shown in Figure 6a–e. At sintering temperatures of 800 °C and 1000 °C, the degree of sintering of the sintered zirconia ceramics is very low due to the relatively low temperatures at this time. This results in looser particles on the surface of the samples. However, as evidenced by the brighter regions depicted in the figure, the surface of the ceramics sintered at these temperatures exhibited varying degrees of agglomeration of particles, with the samples sintered at 1000 °C displaying a larger size and more pronounced degree of agglomeration. This phenomenon can be attributed to the initial stage of sintering, where zirconia ceramics have undergone 2 h of sintering at 800 °C and 1000 °C. In the early stages of sintering, the sintering driving force induces the formation of sintering necks between the ceramic particles and material transport, leading to particle agglomeration. As the sintering temperature increases to 1200 °C, the relative density of the zirconia ceramics is found to be significantly higher in comparison to samples sintered at 800 °C and 1000 °C, despite the presence of a substantial number of pores within the ceramics. As shown in Figure 6f, the average size of the pores inside the zirconia ceramics after sintering at 1200 °C is 101.7 nm, with the largest percentage of the number of pores with the size of 80–100 nm. When the sintering temperatures reached 1400 °C and 1450 °C, the sintered zirconia ceramics exhibited a dense organizational structure with clearly defined grains, and no obvious pores were observed.

**Figure 7:**
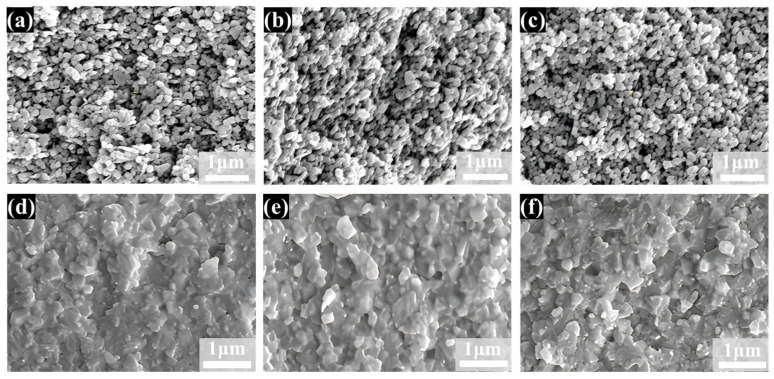
SEM images of zirconia ceramic cross-sections in the green body and at different sintering temperatures: (**a**) green body; (**b**) 800 °C; (**c**) 1000 °C; (**d**) 1200 °C; (**e**) 1400 °C; and (**f**) 1450 °C.

Figure 7 shows SEM images of zirconia ceramic cross-sections in the green body and at different sintering temperatures, showing the microstructural changes with increasing sintering temperature. It can be observed that the cross-sectional organization of the sintered zirconia ceramics is similar to that of the green body with loose particles at sintering temperatures of 800 °C and 1000 °C, as shown in Figure 6a–c. This is also shown in Figure 4 where the relative density of the zirconia ceramics after sintering at these two temperatures is not significantly increased compared to that of the green body. As illustrated in Figure 7d, when the sintering temperature reaches 1200 °C, the SEM image demonstrates a notable transformation in the microstructure of the zirconia ceramics, accompanied by a considerable increase in the degree of densification of the grains. This observation is consistent with the data in Figure 4, where the relative density of the zirconia ceramics exhibits a notable increase between 1000 °C and 1200 °C. Additionally, the figure indicates the persistence of pores in the zirconia ceramics, which is due to the fact that the zirconia ceramic is not fully sintered at this point. As the temperature increases further, the grain profile of the zirconia ceramics becomes more defined, and the fracture mode shifts from predominantly intergranular to a mixed intergranular-transgranular fracture mode, see Figure 7f.

The grain size of ceramic materials can be expressed using the following empirical formula [56]:

(3) $$d^{n}-d_{0}^{n}=kt\cdot \exp\left(-\frac{Q}{RT}\right)$$

In the formula, d is the grain size after growth (nm), $d_{0}$ is the initial grain size (nm), k and t are physical constants related to the specific material, n is the growth exponent, R is the universal gas constant, T is the temperature, and Q is the activation energy. From Equation (3), it can be seen that the grain size increases with the rise in sintering temperature.

**Figure 8:**
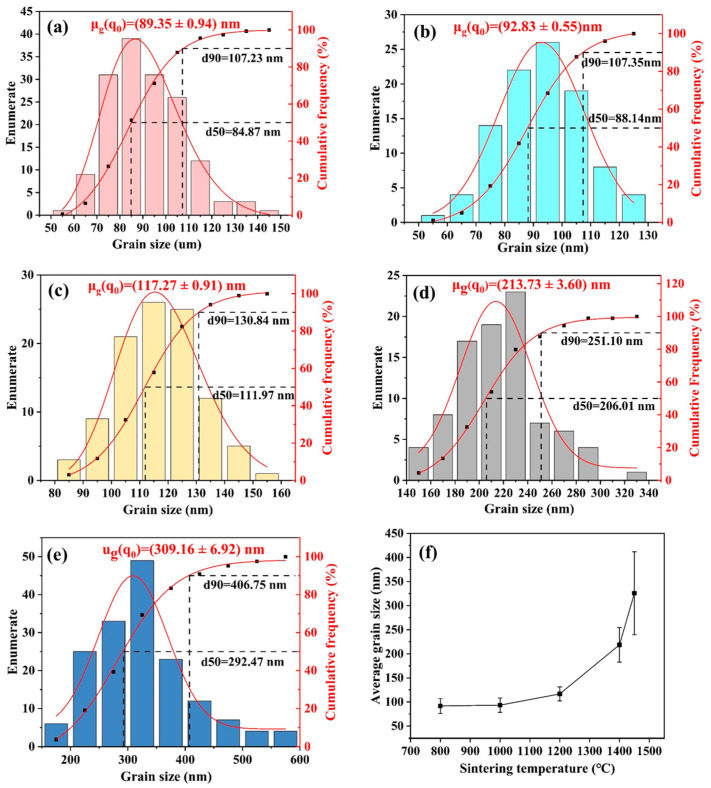
(**a**–**e**) The grain size distribution diagrams of the zirconia ceramic sintered at 800 °C, 1000 °C, 1200 °C, 1400 °C, and 1450 °C, respectively; and (**f**) the average grain sizes of the zirconia ceramic at different sintering temperatures.

Figure 8a–e shows the grain size distribution of zirconia ceramics at different sintering temperatures. The results indicate that the average grain size of zirconia gradually increases with the rise in sintering temperature. The average grain sizes at the five sintering temperatures are 89.35 nm, 92.83 nm, 117.27 nm, 213.73 nm, and 309.16 nm, respectively. The average grain sizes of the samples at sintering temperatures of 800 °C and 1000 °C are quite similar. However, at 800 °C, the most frequent grain size range is 80–90 nm. After sintering at 1000 °C, the most frequent grain size range is 90–100 nm, indicating that the grain size increases with the rise in sintering temperature. When the sintering temperature increases from 1000 °C to 1200 °C, the average grain size increases from 92.83 nm to 117.27 nm, with over 60% of the grain sizes falling between 105 nm and 135 nm. Additionally, in the samples sintered at 800 °C and 1000 °C, 90% of the grain sizes are below 107.35 nm, whereas, with the sintering temperature increased to 1200 °C, this size also increases to 130.84 nm. This trend is consistent with the relationship expressed in Equation (3).

Figure 8f illustrates the trend in the average grain size of zirconia ceramics with increasing sintering temperature. The grain growth rate shows progressive acceleration as sintering temperature rises. Particularly after the sintering temperature attains 1400 °C, where a notable increase in grain growth rate is evident. The grain growth trend observed in this study is broadly consistent with the findings of Liu et al. [57]. The researchers employed DLP to form zirconia ceramics and conducted sintering experiments within the temperature range of 1200–1600 °C. The study demonstrated that the grain growth rate exhibited a gradual acceleration in conjunction with an increase in sintering temperature. The grain size reached 240 ± 60 nm following sintering at 1400 °C. In contrast, the zirconia ceramics prepared by NPJ in the present study showed a smaller grain size (213.73 ± 3.60 nm) after sintering at 1400 °C. This discrepancy is primarily ascribed to the comparatively diminutive powder grain size of the zirconia ceramic feedstock employed in the present study.

**Figure 9:**
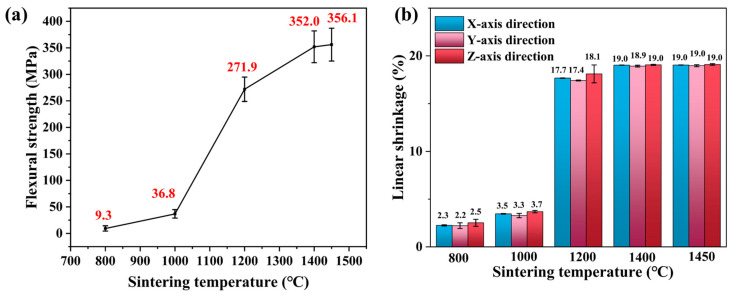
Characteristics of 3Y-TZP 3D-printed sintered body according to sintering temperature: (**a**) flexural strength; (**b**) linear shrinkage in the X, Y and Z directions.

The flexural strength of a material indicates its ability to resist deformation when subjected to a bending load. Figure 9a shows the flexural strength of zirconia ceramics at different sintering temperatures. The results indicate that the flexural strength of sintered zirconia ceramics gradually increases with the rise in sintering temperature. As the sintering temperature increases from 800 °C to 1450 °C, the flexural strength increases from 9.3 MPa to 356.1 MPa. Notably, when the sintering temperature increases from 1000 °C to 1200 °C, the flexural strength of the sintered samples jumps from 36.8 MPa to 271.9 MPa. The growth rate of flexural strength is relatively lower in other sintering temperature ranges. As demonstrated in Figure 9a, in the 1400–1450 °C range, the flexural strength shows merely a 4.1 MPa increase, suggesting the trend is approaching a plateau. In comparison with the study of Zhong et al. in the literature [40], the zirconia ceramics prepared by material jetting exhibited a high relative density of 99.5% after sintering at 1450 °C, while the flexural strength reached 699 ± 104 MPa, which was superior to the 356.1 MPa in the present study. This discrepancy was primarily attributed to the higher degree of densification of the former, which indicated that the sintered body had a lower porosity, thereby enhancing the mechanical properties.

The flexural strength of ceramics is influenced by a combination of grain size and porosity. According to the Hall–Petch relationship, as shown in Equation (4), there is a negative correlation between the yield strength of a material and its grain size. The increase in grain size will lead to a decrease in material strength.

(4) $$\sigma_{y}=\sigma_{0}+\frac{k}{\sqrt{d}}$$

where $\sigma_{y}$ is the yield strength of the material in MPa, $\sigma_{0}$ is the lattice friction stress in N, k is the material constant, dimensionless, and d is the grain size of the material in nm. In addition, the reduced porosity contributes to an increase in the minimum solid area (MSA), which increases the bonding strength between the grains and further improves the flexural strength of the ceramic [58,59].

When the sintering temperature is in the range of 800 °C to 1000 °C, the porosity decreases at a slow rate in conjunction with the increase in density of zirconia ceramics. However, at this temperature, the grain size remains essentially unchanged, resulting in a gradual increase in flexural strength with the reduction in porosity. Upon reaching a sintering temperature of 1000 °C to 1200 °C, the density and grain size of zirconia ceramics exhibit an increase. However, the rate of change in density is markedly higher than that of grain size. At this temperature range, the reduction in porosity plays a dominant role, resulting in a similar trend in flexural strength with relative density. Subsequently, with the further increase in sintering temperature, the increase rate of density decelerates, while the growth rate of grain size markedly accelerates. The effect of grain size on the flexural strength is consequently enhanced. The combined effect of these two factors results in a gradual decrease in the rate of increase in flexural strength. The increase trend of flexural strength begins to level off at 1450 °C. The linear shrinkage of zirconia ceramics in the X, Y and Z directions is demonstrated in Figure 9b. Shrinkage in all three directions is nearly equal across sintering temperatures, indicating high uniformity. This uniform shrinkage behavior helps maintain the dimensional accuracy of fabricated parts.

## 4. Conclusions

This study aims to investigate the effect of sintering temperature on the flexural strength of zirconia ceramics printed by NPJ. By sintering at different temperatures, the effects of sintering temperature on phase composition, microstructure, and flexural strength were examined. This provides guidance for optimizing the sintering process of NPJ-printed zirconia ceramics in the future. Based on the current research results, the following conclusions can be drawn:

1. The NPJ green body is composed of m-ZrO_2_ and t-ZrO_2_. At a sintering temperature of 1000 °C, m-ZrO_2_ has completely transformed into t-ZrO_2_, and only t-ZrO_2_ is observed in samples sintered at higher temperatures.
2. As the sintering temperature increases from 800 °C to 1450 °C, the average grain size of zirconia increases from 89.35 nm to 309.16 nm, while its relative density rises from 55.0% to 98.3%.
3. At sintering temperatures of 800 °C and 1000 °C, the flexural strength of sintered zirconia increases only slightly. When the sintering temperature rises from 1000 °C to 1200 °C, the flexural strength significantly increases from 36.8 MPa to 271.9 MPa, representing 67.8% of the total flexural strength gain. After sintering at 1450 °C, zirconia ceramic achieves a flexural strength of 356.1 MPa. The improvement in zirconia’s flexural strength is primarily attributed to a combination of grain size and porosity.

## Figures and Tables

**Table 1:** The composition of zirconia ceramic slurry and support slurry.

Slurry Type	Ingredient
Zirconia ceramic slurry	Zirconia 40–45 wt.%, ethylene glycol 53–58 wt.%, polyvinyl alcohol polymer and other additives is 1.0–3.0 wt.%
Support slurry	Sodium carbonate 27–32 wt.%, glycol 65–70 wt.%, other additives such as polyphosphate polymers ≤ 2.0 wt.%

## Data Availability

The original contributions presented in this study are included in the article. Further inquiries can be directed to the corresponding authors.
